# Prevalence of Clinically Significant Extraosseous Findings on Unenhanced CT Portions of ^18^F-Fluoride PET/CT Bone Scans

**DOI:** 10.1100/2012/979867

**Published:** 2012-09-10

**Authors:** Chao-Jung Chen, Shih-Ya Ma

**Affiliations:** Department of Nuclear Medicine, Yuan's General Hospital, 162 Cheng-Kung 1st Road, Kaohsiung 802, Taiwan

## Abstract

*Objective*. Due to the frequently interrupted supply of ^99m^Tc-methylene diphosphonate, the use of ^18^F-fluoride positron emission tomography (PET)/computed tomography (CT) has become more popular. The study aims to determine the percentage of extraosseous findings from the unenhanced CT portion of ^18^F-fluoride PET/CT scans. *Materials and Methods*. We retrospectively collected ^18^F-fluoride PET/CT studies between March 2010 and February 2011. The unenhanced CT portions of ^18^F-fluoride PET/CT were reviewed for each patient. Significant extraosseous findings related to malignancy from the unenhanced CT were recorded. *Results*. A total of 158 patients (110 females, 48 males) were included in the study. Clinically significant extraosseous findings from the unenhanced CT were found in 43 patients (27.2%). Previously unknown extraosseous findings were identified in 17 patients (10.8%) after a review of the ^18^F-fluoride PET/CT scan results. Most of the extraosseous findings were small pulmonary metastases or enlarged metastatic lymph nodes. *Conclusion*. It is not rare to identify new clinically significant extraosseous findings from the unenhanced CT of ^18^F-fluoride PET/CT studies. Therefore the clinical management of patients may be altered by the results, and a careful review of the unenhanced CT portion of ^18^F-fluoride PET/CT is mandatory.

## 1. Introduction

^18^F-fluoride is a tracer element for bone scintigraphy that was introduced by Blau and others in the early 1960s. It was approved for clinical use by the U.S. Food and Drug Administration in 1972. However, the relatively high energy of the 511-keV annihilation photons produced by the decay of ^18^F prohibited its widespread use in the era of Anger-type *γ*-cameras suitable for the 140-keV photons of ^99m^Tc. ^99m^Tc-methylene diphosphonate (MDP) was therefore the most suitable technique for whole-body surveys due to its wide availability and consistent low cost [1].

The successful development of positron emission tomography (PET)/computed tomography (CT) and the frequent interruption of ^99m^Tc-MDP supply have led to a renewed interest in the use of ^18^F-fluoride PET/CT to detect bone metastases in cancer patients. Although the CT portion of the scan is mainly used for anatomic localization and attenuation correction, the scan might contain valuable information not shown on the PET image. Clinically significant findings from the unenhanced CT portion of ^18^F-fluorodeoxyglucose (FDG) PET/CT and myocardial perfusion single photon emission computed tomography (SPECT)/CT have been discussed [2–4]. To our knowledge, no information currently exists on the utility of the unenhanced CT portion from ^18^F-fluoride PET/CT. The purpose of this study was to assess the prevalence of clinically significant extraosseous findings from the unenhanced CT portions of ^18^F-fluoride PET/CT.

## 2. Materials and Methods

The study retrospectively included patients with known or suspected malignancy that underwent ^18^F-fluoride PET/CT studies for the detection of bone metastasis either for staging or followup between March 2010 and February 2011. The study protocol was approved by the institutional review board.

### 2.1. ^18^F-Fluoride PET/CT

Each patient was given 370 MBq (10 mCi) of ^18^F-fluoride intravenously. Subsequently, an integrated PET/CT scanner (Biograph; Siemens AG, Berlin, Germany) was used to acquire full body images 1 h after injection. The emission data acquisition time per bed was 3 minutes. The 6-slice CT was acquired using the following scanning parameters: 130 kVp, 95 mA, PITCH: 1.5, slice thickness: 3 mm. No CT contrast agent was administered. Both PET and CT scans were performed for patients under shallow breathing. All patients placed their arms at their sides during the CT acquisition. The images were reconstructed with a standard ordered-subset expectation maximization algorithm.

### 2.2. CT Interpretation

Two experienced board-certified nuclear medicine physicians reviewed the unenhanced CT scans using soft tissue, lung, and bone window settings. All skeletal findings from the unenhanced CT were excluded. Extraosseous findings from the unenhanced CT were considered significant and recorded if they were suspected to be malignant. Other extraosseous findings not related with malignancy, such as renal stone, were judged as nonsignificant and excluded. The clinically significant extraosseous findings from the unenhanced CT of ^18^F-fluoride PET/CT were compared with the previous exams and separated into either previously known or unknown groups. All patients had comparisons with followup imaging studies to confirm the findings from the unenhanced CT of ^18^F-fluoride PET/CT and to check for the existence of any missed lesions.

## 3. Results

A total of 158 patients were recruited in the study. There were 110 female patients and 48 male patients. The average age was 57 years old (range: 31–84 years old). These patients had diverse malignancies including 92 breast cancers, 16 hepatocellular carcinomas (HCC), 9 lung cancers, 7 nasopharyngeal carcinomas, 4 buccal cancers, 4 colon cancers, 4 esophageal cancers, 4 prostate cancers, 4 tongue cancers, and 14 other forms of cancer.

**Figure 1:**
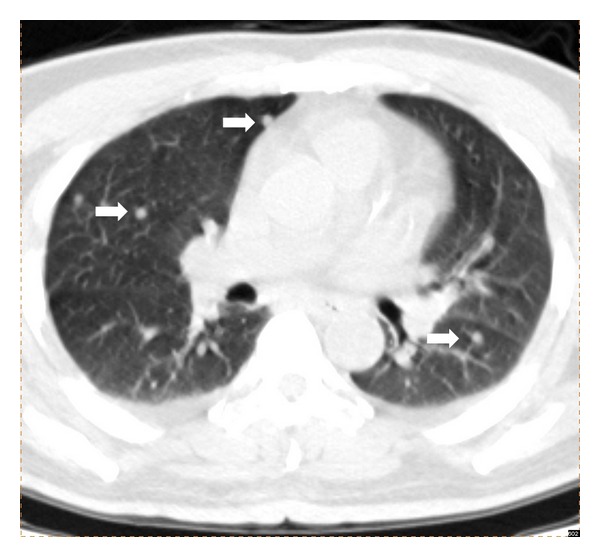
CT image from a 48-year-old male patient diagnosed with hepatocellular carcinoma. The followup unenhanced CT of ^18^F-fluoride PET/CT, in the lung window, revealed numerous tiny pulmonary nodules in the bilateral lungs (arrows). The largest nodule was 0.6 cm in size and none of these nodules were visualized by concurrent chest X-ray.

**Figure 2:**
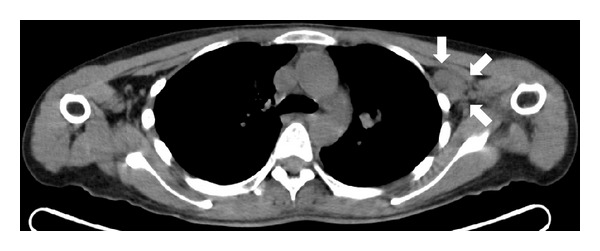
CT image from a 34-year-old female patient diagnosed with cancer in the left breast. The patient had surgery and regular followup care for two years. The followup unenhanced CT of ^18^F-fluoride PET/CT showed multiple enlarged lymph nodes including the left axillary lymph node (arrows, 2.8 × 1.7 cm in size). ^18^F-FDG PET/CT six days later confirmed the presence of these metastatic lymph nodes.

**Figure 3:**
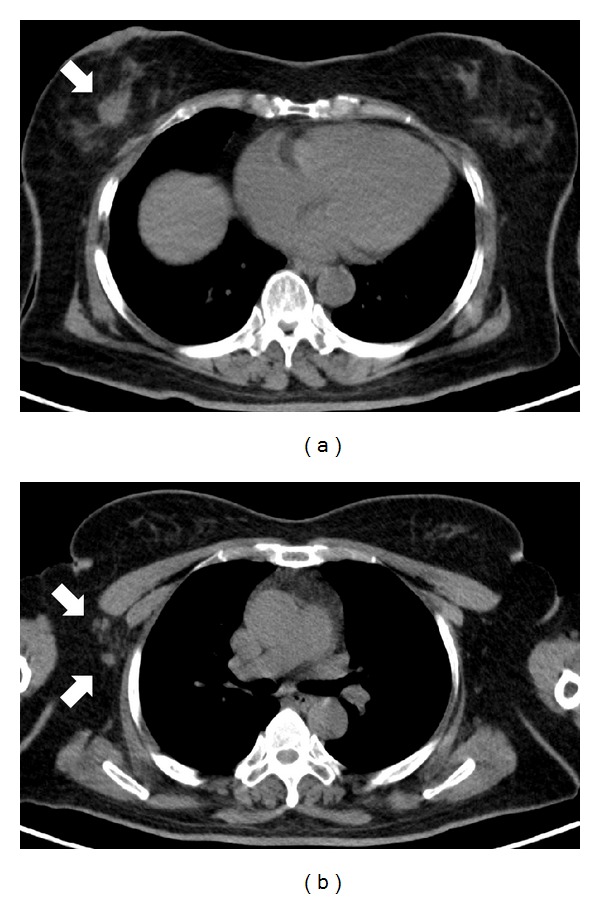
Incidental right breast mass (a) arrow, 2.7 × 1.7 cm in size) and several right axillary lymph nodes (b) arrows, the largest one is 1.0 × 0.8 cm in size) in a 59-year-old female with history of endometrial cancer were found by followup unenhanced CT of ^18^F-fluoride PET/CT. The biopsy for the right breast tumor confirmed the presence of invasive ductal carcinoma.

43 patients (27.2%) demonstrated clinically significant extraosseous findings from the unenhanced CT of ^18^F-fluoride PET/CT. After excluding previously known cases, 17 patients (10.8%) showed clinically significant new extraosseous findings (Table 1). Small pulmonary metastases were identified in 10 patients (Figure 1). Enlarged metastatic lymph nodes were found in 6 patients (Figure 2). Incidental primary malignancies, including lung and breast cancer (Figure 3), were discovered in two patients.

Unenhanced CT from ^18^F-fluoride PET/CT was unable to identify HCC tumors in 9 of 16 patients with either primary or recurrent hepatic tumors detected with concurrent CT or magnetic resonance imaging (MRI). Further retrospective review of the CT results only identified obscure images.

## 4. Discussion

^18^F-fluoride PET has been proven to be more useful than ^99m^Tc-MDP scintigraphy for detection of bone metastasis in a variety of malignancies [5, 6]. With the aid of CT, ^18^F-fluoride PET/CT is better than ^18^F-fluoride PET alone and is more accurate than ^99m^Tc-MDP scintigraphy when compared to SPECT/CT [7–9]. Although the original role of CT in ^18^F-fluoride PET/CT is for identifying anatomic landmarks, it also provides a large amount of diagnostic information. Our study showed that most extraosseous findings occurred in the lungs. Given that low dose CT without contrast enhancement has been used widely in lung cancer screening for many years [10, 11], it is not surprising that the unenhanced CT in the PET/CT can also detect pulmonary lesions. These previously unknown small pulmonary metastases detected by the unenhanced CT of PET/CT could influence the future clinical management of these patients. Chest X-rays which are routinely used for followup care in lung cancer patients are not efficient at detecting small pulmonary nodules. In patients with HCC, the followup abdominal CT only images the lower part of the lung. Furthermore, the use of an additional low-dose CT scan during maximal inspiration after a PET/CT scan has been suggested [12]. However, based on our experience, the unenhanced CT portion of ^18^F-fluoride PET/CT with shallow breathing can detect the same pulmonary lesions as conventional contrast enhanced chest CT. The second most common extraosseous findings identified in this study were enlarged metastatic lymph nodes. Based on these results, contrast enhancement is not necessary for detection of metastatic lymph nodes [13]. Careful CT image analysis makes identifying metastatic lymph nodes possible. Primary malignancies, including lung and breast cancer, were incidentally observed in two patients. These findings suggest the possibility of using unenhanced CT of ^18^F-fluoride PET/CT for early detection for primary malignancy. The above-mentioned clinically significant extraosseous findings from the unenhanced CT of ^18^F-fluoride PET/CT show that this test can be used to identify previously unknown malignancies, and these results urge further study. Identifying additional lesions using this technique may lead to changes in the therapeutic management of patients.

Although unenhanced CT may provide diagnostic information, there are limitations to this test. Without contrast enhancement, the CT poorly detects lesions in the solid organs such as the liver [14]. In our study, no hepatic tumors were found using unenhanced CT of PET/CT. Solid organ lesions missed by CT could be addressed by using abdominal ultrasonography as a complementary tool. The addition of contrast enhancement in ^18^F-FDG PET/CT has been widely discussed and can contribute additional information [15]. Conversely, no studies on contrast enhancement have been conducted with the ^18^F-fluoride PET/CT because CT is primarily used for lesion localization. Future use of contrast enhancement may permit the identification of previously undetectable liver lesions, thus reducing the necessity of abdominal ultrasonography.

The present study using unenhanced CT to image extraosseous lesions has limitations. First, it was a retrospective study and the ^18^F-fluoride PET/CT was only performed when ^99m^Tc-MDP was unavailable. Therefore, a potential bias in patient selection is possible. Second, with the exception of breast cancer patients, the total number of patients with other forms of cancer is small. The actual percentage and importance of clinically significant extraosseous findings from unenhanced CT in other kinds of malignancy are uncertain. A large prospective study for a specific malignancy comparing CT with conventional radiological studies is needed. Finally, this study could be criticized because the unenhanced CT portion of PET/CT was not reviewed by radiologists or nuclear medicine physicians with additional CT training [2, 3, 16, 17]. However, in our study no potentially visible lesions were missed in the unenhanced CT portion of ^18^F-fluoride PET/CT when compared with followup studies such as contrast enhanced CT, MRI, or ^18^F-FDG PET/CT. Moreover, most nuclear medicine physicians also review the unenhanced CT in their daily practice of analyzing ^18^F-FDG PET/CT. Nuclear medicine physicians are capable of interpreting the unenhanced CT portion of ^18^F-fluoride PET/CT [4].

The frequent shortages and uncertain future supply of ^99^Mo may be a potential crisis that reduces the consistent use of ^99m^Tc-MDP bone scintigraphy. In contrast, the supply of ^18^F-fluoride is increasing because of its wide spread use in cyclotrons. Although ^18^F-fluoride is currently more expensive than ^99m^Tc-MDP, the price differential could decrease due to the increasing availability of cyclotrons. In addition, regular followup for cancer patients commonly includes basic tests such as chest X-ray and abdominal ultrasonography. ^18^F-fluoride PET/CT with contrast enhancement can provide information for the entire skeletal system. This information cannot easily be obtained with other radiological images and can reduce the cost associated with chest X-rays and abdominal ultrasonography. The CT of ^18^F-fluoride PET/CT can detect pulmonary nodules less than 1 cm in diameter, which are difficult to verify by chest X-ray, and has the ability to detect enlarged lymph nodes through a whole body survey. By using contrast enhancement, potential lesions in solid organs such as the liver may also be detected. Contrast enhanced CT may lead to the replacement of abdominal ultrasonography. In the future, contrast enhanced ^18^F-fluoride PET/CT may be used in the followup of cancer patients. However, further studies are needed to determine whether this approach is cost-effective.

## 5. Conclusion

According to our preliminary data, it is not uncommon to locate previously undetected, clinically significant, extraosseous findings using unenhanced CT in the ^18^F-fluoride PET/CT. These new findings may have a great impact on the therapeutic planning and treatment of patients. Routinely and carefully reviewing the unenhanced CT portion of ^18^F-fluoride PET/CT is necessary.

## Figures and Tables

**Table 1:** Clinically significant extraosseous findings on CT of ^18^F-fluoride PET/CT.

Patient no.	Age (y)	Sex	Type of cancer	Incidental findings by unenhanced CT
1	54	F	Breast cancer	Chest wall recurrence
2	46	F	Breast cancer	Pulmonary metastases
3	71	F	Breast cancer	Pulmonary metastases
4	34	F	Breast cancer	Axillary metastatic lymph nodes
5	68	F	Breast cancer	Pulmonary metastases and axillary metastatic lymph nodes
6	34	F	Breast cancer	Axillary metastatic lymph nodes
7	47	F	Breast cancer	Axillary metastatic lymph nodes
8	50	F	Breast cancer	Pulmonary metastases
9	61	F	Hepatocellular carcinoma	Pulmonary metastases
10	68	M	Hepatocellular carcinoma	Pulmonary metastases
11	50	M	Hepatocellular carcinoma	Pulmonary metastases
12	48	M	Hepatocellular carcinoma	Pulmonary and adrenal metastases
13	51	M	Buccal cancer	Cervical metastatic lymph nodes
14	70	F	Colon cancer	Pulmonary metastases
15	59	F	Endometrical cancer	Primary breast cancer with axillary metastatic lymph nodes
16	36	F	Ovarian cancer	Metastatic tumor of abdominal wall
17	45	M	Tongue cancer	Primary lung cancer with ipsilateral pulmonary metastases

PET: positron emission tomography; CT: computed tomography.

## References

[1] Grant FD, Fahey FH, Packard AB, Davis RT, Alavi A, Treves ST (2008). Skeletal PET with ^18^F-fluoride: applying new technology to an old tracer. *Journal of Nuclear Medicine*.

[2] Osman MM, Cohade C, Fishman EK, Wahl RL (2005). Clinically significant incidental findings on the unenhanced CT portion of PET/CT studies: frequency in 250 patients. *Journal of Nuclear Medicine*.

[3] Husmann L, Tatsugami F, Aepli U (2009). Prevalence of noncardiac findings on low dose 64-slice computed tomography used for attenuation correction in myocardial perfusion imaging with SPECT. *International Journal of Cardiovascular Imaging*.

[4] Goetze S, Pannu HK, Wahl RL (2006). Clinically significant abnormal findings on the “nondiagnostic” CT portion of low-amperage-CT attenuation-corrected myocardial perfusion SPECT/CT studies. *Journal of Nuclear Medicine*.

[5] Schirrmeister H, Guhlmann A, Elsner K (1999). Sensitivity in detecting osseous lesions depends on anatomic localization: planar bone scintigraphy versus ^18^F PET. *Journal of Nuclear Medicine*.

[6] Schirrmeister H, Glatting G, Hetzel J (2001). Prospective evaluation of the clinical value of planar bone scans, SPECT, and ^18^F-labeled NaF PET in newly diagnosed lung cancer. *Journal of Nuclear Medicine*.

[7] Even-Sapir E, Metser U, Flusser G (2004). Assessment of malignant skeletal disease: initial experience with ^18^F-fluoride PET/CT and comparison between ^18^F-fluoride PET and ^18^F-fluoride PET/CT. *Journal of Nuclear Medicine*.

[8] Even-Sapir E, Metser U, Mishani E, Lievshitz G, Lerman H, Leibovitch I (2006). The detection of bone metastases in patients with high-risk prostate cancer: ^99m^Tc-MDP planar bone scintigraphy, single- and multi-field-of-view SPECT, ^18^F-fluoride PET, and ^18^F-fluoride PET/CT. *Journal of Nuclear Medicine*.

[9] Yen RF, Chen CY, Cheng MF (2010). The diagnostic and prognostic effectiveness of F-18 sodium fluoride PET-CT in detecting bone metastases for hepatocellular carcinoma patients. *Nuclear Medicine Communications*.

[10] Veronesi G, Bellomi M, Mulshine JL (2008). Lung cancer screening with low-dose computed tomography: a non-invasive diagnostic protocol for baseline lung nodules. *Lung Cancer*.

[11] MacMahon H, Austin JHM, Gamsu G (2005). Guidelines for management of small pulmonary nodules detected on CT scans: a statement from the Fleischner Society. *Radiology*.

[12] Kuehl H, Veit P, Rosenbaum SJ, Bockisch A, Antoch G (2007). Can PET/CT replace separate diagnostic CT for cancer imaging? Optimizing CT protocols for imaging cancers of the chest and abdomen. *Journal of Nuclear Medicine*.

[13] Rodríguez-Vigil B, Gómez-León N, Pinilla I (2006). PET/CT in lymphoma: prospective study of enhanced full-dose PET/CT versus unenhanced low-dose PET/CT. *Journal of Nuclear Medicine*.

[14] Gollub MJ, Hong R, Sarasohn DM, Akhurst T (2007). Limitations of CT during PET/CT. *Journal of Nuclear Medicine*.

[15] Antoch G, Freudenberg LS, Beyer T, Bockisch A, Debatin JF (2004). To enhance or not to enhance? ^18^F-FDG and CT contrast agents in dual-modality ^18^F-FDG PET/CT. *Journal of Nuclear Medicine*.

[16] Schöder H, Yeung HWD, Larson SM (2005). CT in PET/CT: essential features of interpretation. *Journal of Nuclear Medicine*.

[17] Coleman RE, Delbeke D, Guiberteau MJ (2005). Concurrent PET/CT with an integrated imaging system: intersociety dialogue from the joint working group of the American College of Radiology, the Society of Nuclear Medicine, and the Society of Computed Body Tomography and Magnetic Resonance. *Journal of Nuclear Medicine*.

